# Comprehensive Endoscopic Management of Superficial Pharyngeal Cancer – From Detection and Diagnosis to Treatment and Surveillance

**DOI:** 10.1002/deo2.70180

**Published:** 2025-08-11

**Authors:** Daisuke Kikuchi, Yugo Suzuki, Satoshi Yamashita, Yorinari Ochiai, Shu Hoteya

**Affiliations:** ^1^ Department of Gastroenterology Toranomon Hospital Kajigaya Kanagawa Japan; ^2^ Diagnostic and Therapeutic Gastrointestinal Endoscopy Clinic Kanagawa Japan; ^3^ Department of Gastroenterology Toranomon Hospital Tokyo Japan

**Keywords:** endoscopic submucosal dissection, image‐enhanced endoscopy, lymph node metastasis, magnifying endoscopy, pharyngeal cancer

## Abstract

Traditionally, pharyngeal cancer was detected at an advanced stage, as examinations were usually performed only after symptoms such as pain or dysphagia appeared. Consequently, it was considered a malignancy with a poor prognosis. However, recent advances in image‐enhanced endoscopy (IEE) have facilitated the early detection of superficial pharyngeal cancers. The combination of IEE and magnifying endoscopy enables both the detection and detailed characterization of lesions, including assessment of malignancy and invasion depth. Due to the anatomically complex structure of the pharyngolaryngeal region, en bloc resection using snare‐based endoscopic mucosal resection has been difficult. However, the development of endoscopic submucosal dissection (ESD) and endoscopic laryngopharyngeal surgery has made en bloc resection technically possible. Successful pharyngeal ESD requires careful planning, including consideration of intubation route, laryngoscope positioning, and choice of devices. Tumor thickness ≥1000 µm and positive lymphovascular invasion are pathological risk factors for lymph node metastasis. However, no consensus exists regarding the need for additional adjuvant chemotherapy. After pharyngeal ESD, close follow‐up is essential, focusing on lymph node metastasis and metachronous cancers. Lymph node metastasis may require dissection or radiotherapy, while metachronous lesions can often be treated endoscopically. A multidisciplinary approach is essential for effective management of superficial pharyngeal cancer.

## 1 Introduction

In recent years, advances in image‐enhanced endoscopy (IEE) and magnifying endoscopy have led to an increased detection of superficial pharyngeal cancers during routine gastrointestinal endoscopic examinations. Despite this progress, many cases are still diagnosed at an advanced stage. To achieve early detection, endoscopists must recognize and apply certain key tips and practical points specific to the pharyngolaryngeal region. Although the endoscopic appearance of pharyngeal squamous cell carcinoma largely resembles that of esophageal squamous cell carcinoma, there are distinct differences due to the unique anatomical features of the pharyngolaryngeal region.

Therapeutic approaches have also advanced considerably in recent years. Transoral procedures such as endoscopic submucosal dissection (ESD) and endoscopic laryngopharyngeal surgery (ELPS) are increasingly being adopted in clinical practice. However, challenges remain, including the requirement for specialized equipment and the need for close cooperation with otolaryngologists.

Although several pathological risk factors for lymph node metastasis have been reported, there is still no established consensus on how they should be incorporated into clinical decision‐making. Moreover, the incidence of metachronous cancers in the pharyngeal region is relatively high compared to other parts of the gastrointestinal tract, emphasizing the importance of careful long‐term surveillance.

In this article, we review the current status of endoscopic practice for superficial pharyngeal cancer, focusing on detection, diagnosis, treatment, and surveillance.

## 2 Detection of Superficial Pharyngeal Cancer

To increase the detection rate of superficial pharyngeal cancer, it is essential to identify high‐risk patients and perform appropriate endoscopic examinations tailored to these individuals.

### 2.1 Risk Factors for Pharyngeal Cancer

The prevalence of pharyngeal cancer is relatively low, and endoscopic observation of the pharynx can cause discomfort for patients. Therefore, routine pharyngeal screening for all individuals is not recommended. To improve the detection of pharyngeal cancer, it is essential to identify and focus on high‐risk patients [1, 2].

Alcohol consumption and tobacco smoking are major risk factors for pharyngeal cancer. In particular, individuals who experience facial flushing after consuming small amounts of alcohol are considered to be at high risk and should undergo careful endoscopic examination [3]. Other reported risk factors include a history of squamous cell carcinoma in the pharynx or esophagus, low intake of green and yellow vegetables, and low body mass index (BMI).

### 2.2 Preparation before Endoscopic Examination

Pharyngeal endoscopic observation often induces a strong gag reflex; therefore, appropriate pharyngeal anesthesia and analgesia are essential. In our practice, we apply xylocaine viscous twice, followed by xylocaine spray immediately before the examination. Additionally, 35 mg of intravenous pethidine hydrochloride is administered for analgesia. Sedatives such as midazolam or propofol are not recommended, as they impair the patient's ability to vocalize or perform the Valsalva maneuver, which is important during pharyngeal examination. A randomized controlled trial by Yamazaki et al. also reported a significantly higher detection rate of lesions in the pethidine group [4].

### 2.3 Endoscopic Observation Technique

In high‐risk patients, endoscopic observation begins with inspection of the oral cavity. Because the use of a mouthpiece obstructs visualization of this area, it should be performed without one. Initially, the patient is instructed to curl the tongue upward and press the tip against the hard palate. Then, the patient is asked to touch the tip of the tongue to the inner surfaces of the left and right cheeks. If the patient has difficulty following these instructions, practicing in advance with a hand mirror can improve cooperation and visualization.

**FIGURE 1:**
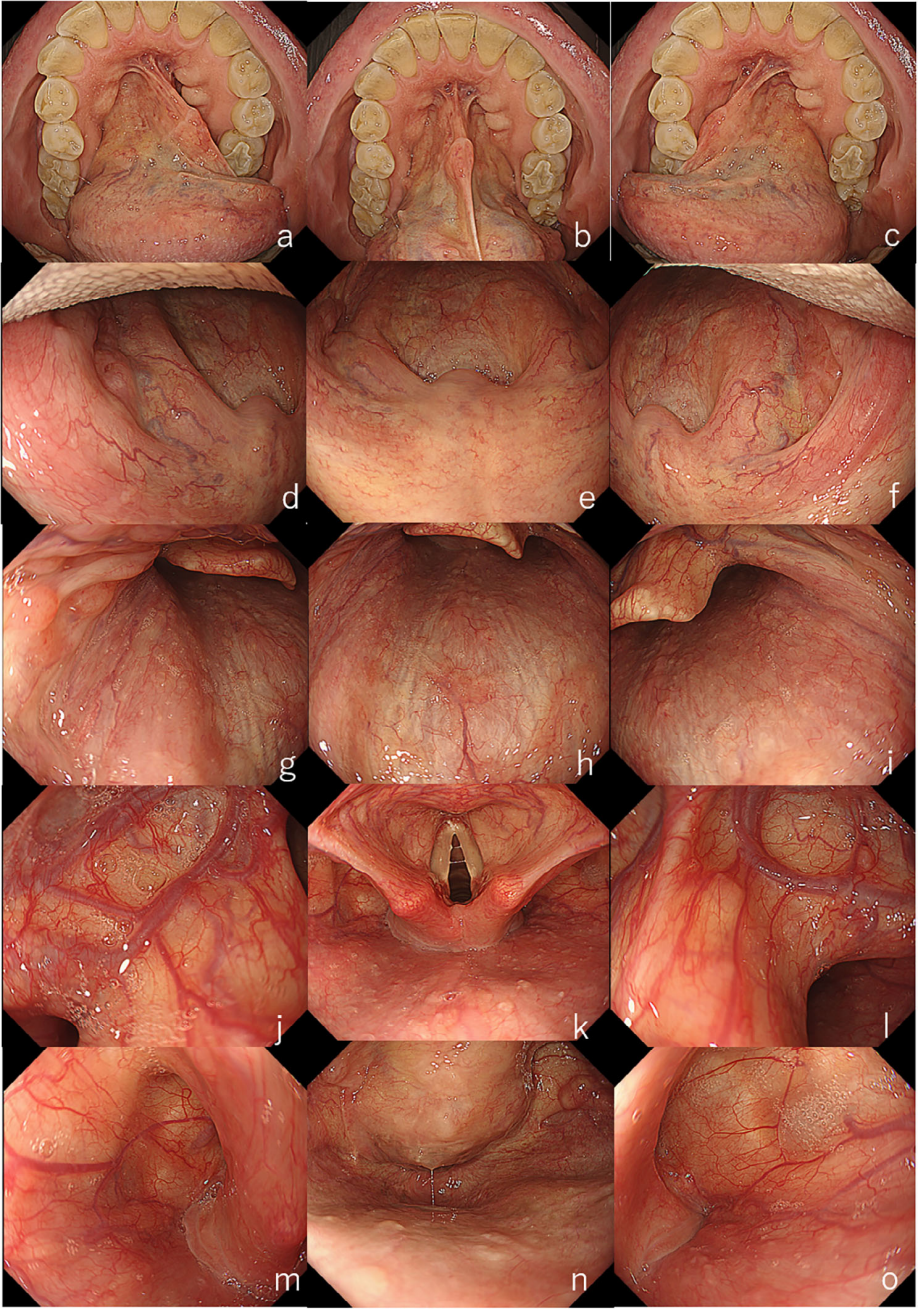
Endoscopic observation of the oral cavity, pharynx, and larynx. (a) Left side of the tongue; (b) Floor of the mouth; (c) Right side of the tongue; (d) Left palatal arch; (e) Soft palate and uvula; (f) Right palatal arch; (g) Left lateral wall of the oropharynx; (h) Posterior wall of the oropharynx; (i) Right lateral wall of the oropharynx; (j) Left vallecula; (k) Larynx; (l) Right vallecula; (m) Left piriform sinus of the hypopharynx; (n) Posterior wall and cricoid area of the hypopharynx; o: Right piriform sinus of the hypopharynx.

After completing the oral cavity observation, a mouthpiece is inserted, and systematic examination begins [5]. The hard and soft palate, uvula, and bilateral palatine arches are observed first, followed by the posterior and lateral walls of the oropharynx. The endoscope is then advanced slowly with upward angulation to visualize the larynx and vocal cords, as well as the bilateral pyriform sinuses. Asking the patient to vocalize at this stage helps close the vocal cords and expand the pyriform sinuses, facilitating observation. The vallecula is then examined bilaterally (Figure 1).

**FIGURE 2:**
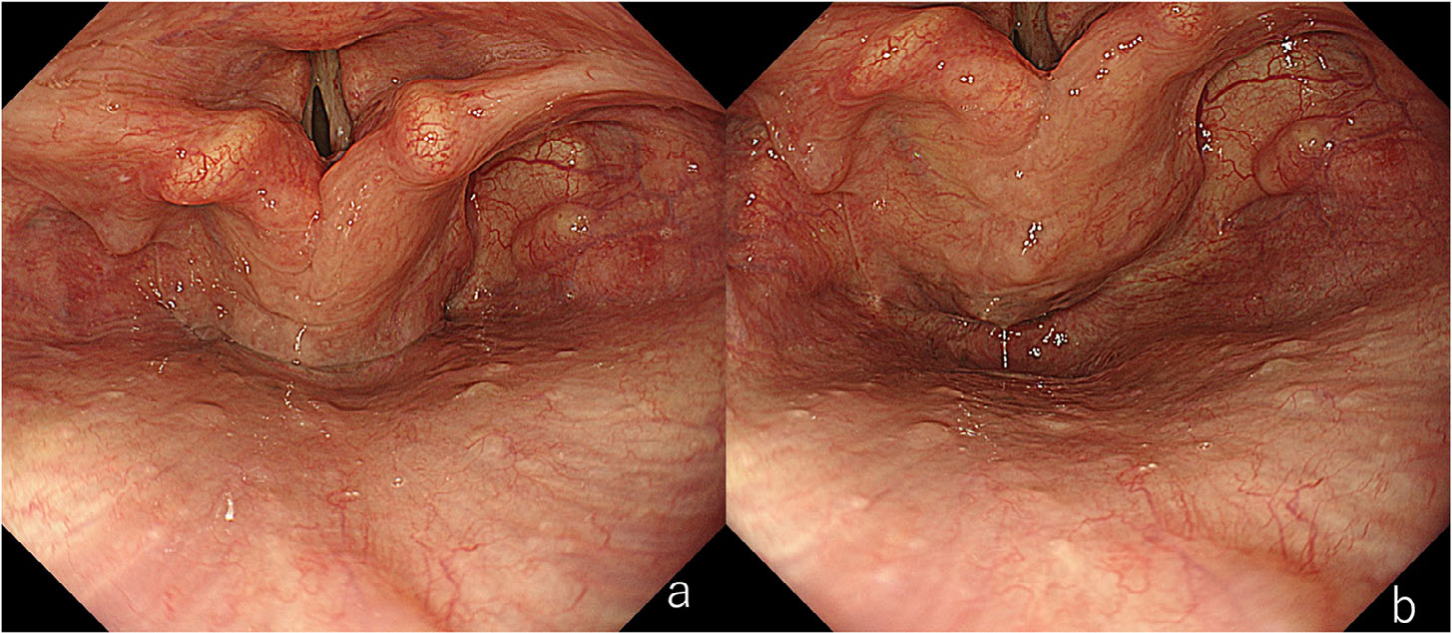
Case undergoing the Valsalva maneuver. (a) During phonation. The posterior wall of the hypopharynx and the cricoid area cannot be observed even during phonation. (b) During the Valsalva maneuver. The larynx elevates, facilitating better observation.

Finally, the Valsalva maneuver is performed. In our experience, this maneuver improves visibility of the posterior cricoid area in approximately 70% of cases. Various methods have been proposed, including transnasal endoscopy, observation without a mouthpiece, and the use of a smaller mouthpiece [6, 7, 8, 9, 10]. We have reported a method using a dedicated mouthpiece (Valsamouth, VM‐001; SB‐Kawasumi Laboratories, Inc., Tokyo, Japan) for performing the Valsalva maneuver during gastrointestinal endoscopy. This technique allows the use of magnifying endoscopy, enabling simultaneous qualitative assessment (Figure 2).

## 3 Endoscopic Diagnosis of Pharyngeal Cancer

The endoscopic appearance of superficial pharyngeal and laryngeal cancers closely resembles that of superficial esophageal squamous cell carcinoma [11]. Under white light endoscopy (WLE), reddish areas, surface irregularity, and keratosis should be carefully evaluated. Loss of visibility of the normal subepithelial vascular pattern is also an important finding. With magnifying IEE, lesions typically appear as brownish areas due to increased density of intrapapillary capillary loops and enhanced background coloration [12]. There is no consensus on whether initial observation should be performed with WLE or IEE. However, a multicenter randomized controlled trial reported that detection rates are significantly higher when magnifying IEE is used, particularly for small lesions that may be missed under WLE [13].

As with other gastrointestinal cancers, assessing the depth of invasion prior to treatment is critical in pharyngeal cancer. Histologically, the pharynx lacks a muscularis mucosae and consists of epithelium, subepithelial layer, and muscular layer. Therefore, the depth of invasion in superficial pharyngeal cancer is expressed as tumor thickness from the surface to the deepest point. Pathologically, lesions are classified as either carcinoma in situ (CIS) or subepithelial invasive carcinoma. The subepithelial invasive carcinomas are further subdivided based on whether the tumor thickness is ≥1000 µm or not. Subepithelial invasive carcinomas with ≥1000 µm thickness are considered to have a higher risk of lymph node metastasis [14].

We have reported that IEE allows assessment of vascular architecture and can be used for depth diagnosis, employing the Japan Esophageal Society classification originally developed for esophageal cancer. Recently, a diagnostic algorithm has been proposed based on both gross morphology under WLE and microvascular patterns under magnifying IEE [15, 16, 17, 18, 19].

**FIGURE 3:**
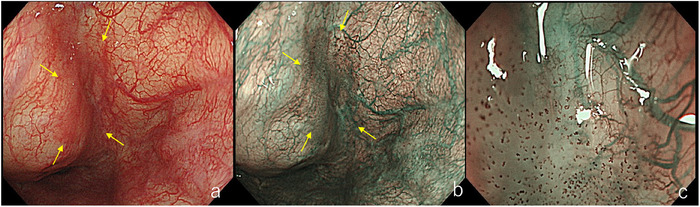
Lesion with Type B1 vessels. (a) Slight erythema is observed in the right piriform sinus of the hypopharynx (yellow arrow). (b) The lesion appears as a brownish area (yellow arrow). (c) Dot‐like vessels with preserved loop structures are proliferating, consistent with Type B1 vessels.

**FIGURE 4:**
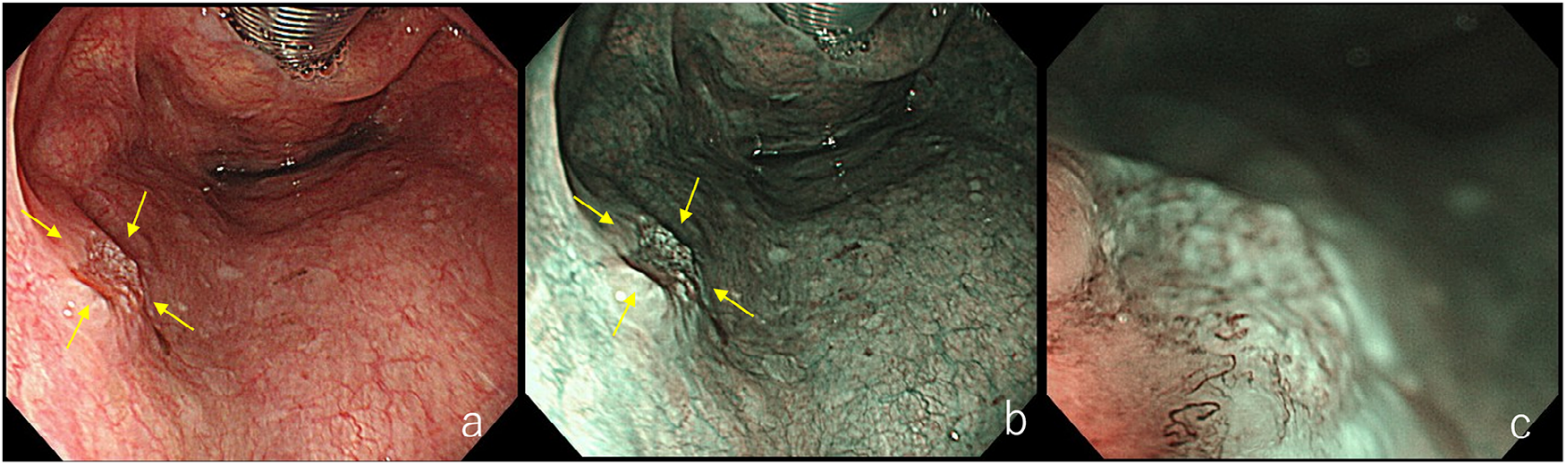
Lesion with Type B2 vessels. (a) A depressed lesion is seen on the left lateral wall of the oropharynx (yellow arrow). (b) The margin appears whitish due to keratinization, and the center is recognized as a brownish area (yellow arrow). (c) Loss of loop structure and presence of meandering vessels are observed, indicating Type B2 vessels.

**FIGURE 5:**
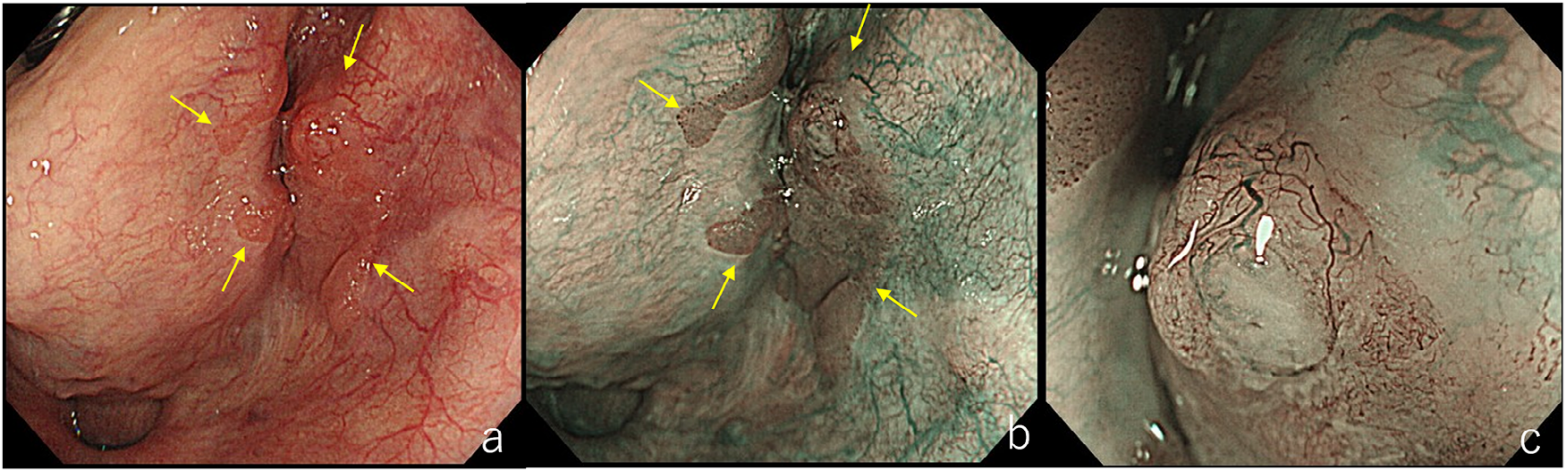
Lesion with Type B3 vessels. (a) Slight erythema is seen in the right piriform sinus of the hypopharynx (yellow arrow). (b) The lesion appears as a brownish area (yellow arrow). (c) Prominent dilated vessels are observed on the elevated part, consistent with Type B3 vessels.

Lesions with a 0‐I gross appearance or type B3 vessels are judged to have a tumor thickness of ≥1000 µm. In contrast, flat 0‐IIb lesions or those with type B1 vessels are typically diagnosed as CIS. Lesions with type B2 vessels are classified as subepithelial invasive carcinoma, and among these, if the gross appearance is 0‐IIa with type B2 vessels, the lesion is considered to have a tumor thickness of ≥1000 µm (Figures 3, 4, 5).

## 4 Endoscopic Treatment of Superficial Pharyngeal Cancer

Endoscopic treatment options for superficial pharyngeal cancer include ESD and ELPS [20, 21, 22, 23]. Each technique has its own advantages and limitations, and treatment should be performed using the method with which the endoscopist and institution are most experienced.

### 4.1 Indications for ESD

ESD is indicated for lesions that are diagnosed as carcinoma by endoscopic and pathological evaluation, with tumor invasion confined to the epithelium or subepithelial layer. There are no strict limitations regarding lesion size or location for ESD. However, the potential impact of post‐ESD scarring on swallowing and phonation should be carefully considered. For large lesions located in the bilateral piriform sinuses, it may be preferable to perform the resection in two separate sessions.

In principle, ESD is performed when there is no evidence of lymph node metastasis prior to treatment. However, in certain cases where lymph node metastasis is suspected or confirmed, ESD may still be performed as a local treatment. In such instances, the decision should not be made solely by the endoscopist but through a multidisciplinary discussion involving otolaryngologists, gastrointestinal surgeons, radiation oncologists, and medical oncologists.

### 4.2 Preoperative Evaluation for ESD

Before performing pharyngeal ESD, we routinely assess the tumor extent and staging using magnifying endoscopy, contrast‐enhanced computed tomography (CT) from the neck to the abdomen, and cervical ultrasonography. When further clarification is needed, positron emission tomography–CT (PET‐CT) is additionally performed.

Pharyngeal ESD is carried out under general anesthesia in all cases. We routinely perform electrocardiography and pulmonary function tests, and cardiac echocardiography is added when indicated. If abnormalities are detected, consultation with cardiology or respiratory medicine specialists is arranged.

Because pharyngeal ESD may affect functions such as swallowing and phonation, preoperative evaluation and discussion with an otolaryngologist are recommended when necessary.

### 4.3 ESD Procedures in the Pharynx

The most critical aspect of pharyngeal ESD is the securement of an adequate working space. Under general anesthesia, the tongue base tends to fall posteriorly, narrowing the pharyngeal space. To create sufficient space, a curved rigid laryngoscope is used to elevate the larynx. Depending on the location of the lesion, the intubation route, position of the laryngoscope tip, and operating endoscopist are determined accordingly.

**FIGURE 6:**
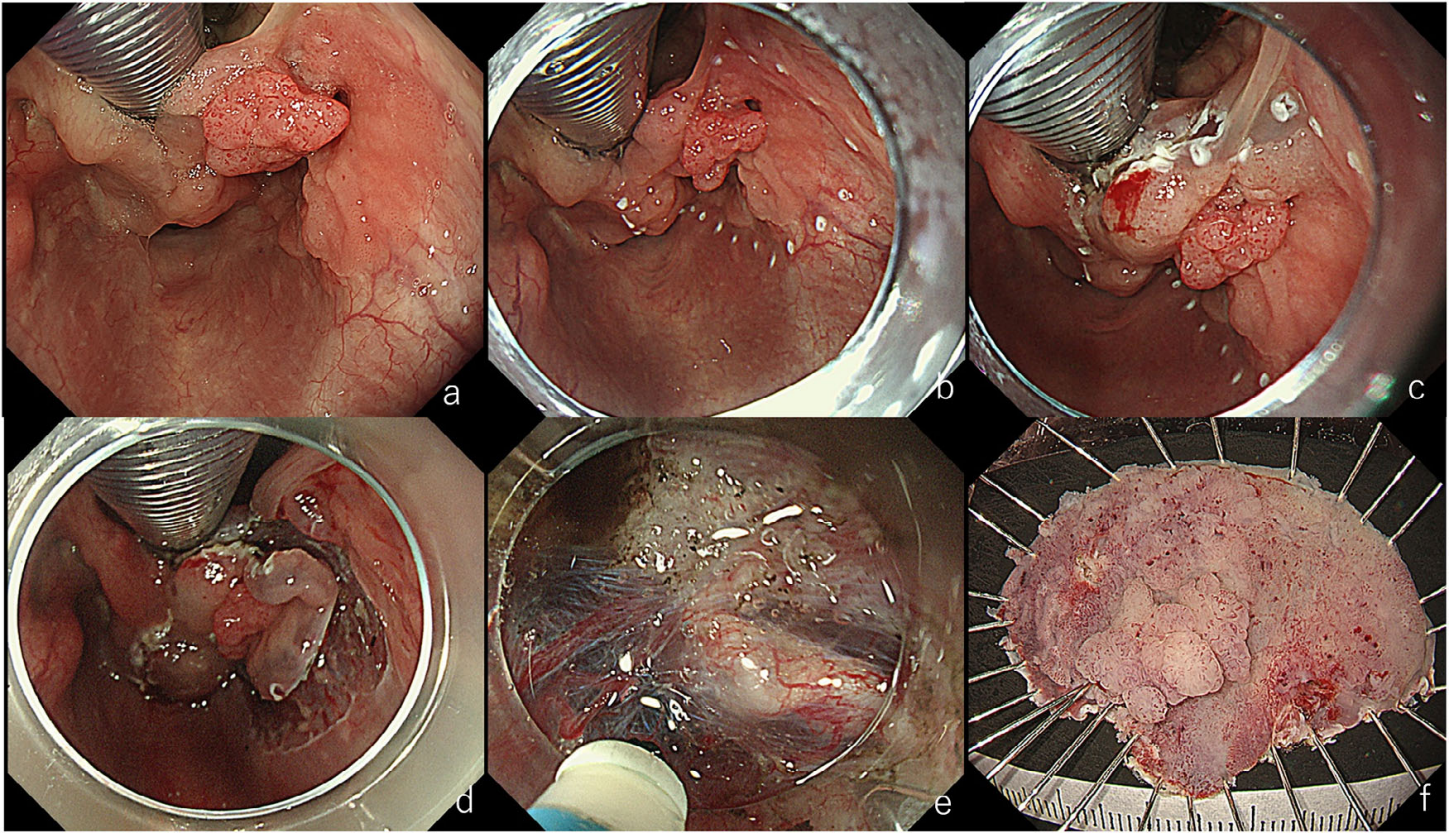
Endoscopic submucosal dissection (ESD) for a lesion in the right piriform sinus of the hypopharynx. (a) After laryngeal elevation. Oral intubation was performed, and the tip of the laryngoscope was positioned in front of the vocal cords to lift the larynx. (b) During marking. Proximity to the aryepiglottic fold was difficult due to the intubation tube. (c) Incision of the aryepiglottic fold. An initial incision was made at this site. (d) After circumferential incision. (e) During dissection. Traction with a laryngeal forceps was applied to better visualize the subepithelial tissue. (f) Resected specimen. En bloc resection was achieved. Pathology revealed squamous cell carcinoma, 42×38 mm, tumor thickness 4800 µm, ly0, v1, HMX, VM0.

For lesions located on the posterior or lateral walls of the oropharynx or within the bilateral pyriform sinuses of the hypopharynx, oral intubation is performed under general anesthesia. The laryngoscope tip is positioned anterior to the vocal cords to elevate the larynx and expose the lesion. Once the larynx is elevated and the lesion identified, iodine staining is applied. Markings are made around the lesion, followed by local injection, mucosal incision, and submucosal dissection. During dissection, traction is applied using a laryngeal forceps to expose the appropriate subepithelial layer [24] (Figure 6).

The posterior oropharyngeal wall has a particularly thin subepithelial layer, and because the dissection angle is perpendicular when approaching orally, transnasal insertion of an ultrathin endoscope is advantageous [25, 26]. This allows for a horizontal approach to the dissection layer, minimizing interference with laryngeal instruments. Recently, some reports have described the use of the water pressure method—originally developed for duodenal ESD—in the pharynx to improve visualization of the dissection layer [27]. However, due to an increased risk of aspiration, caution is advised. After lesion removal, extubation is performed only after confirming the absence of laryngeal edema.

**FIGURE 7:**
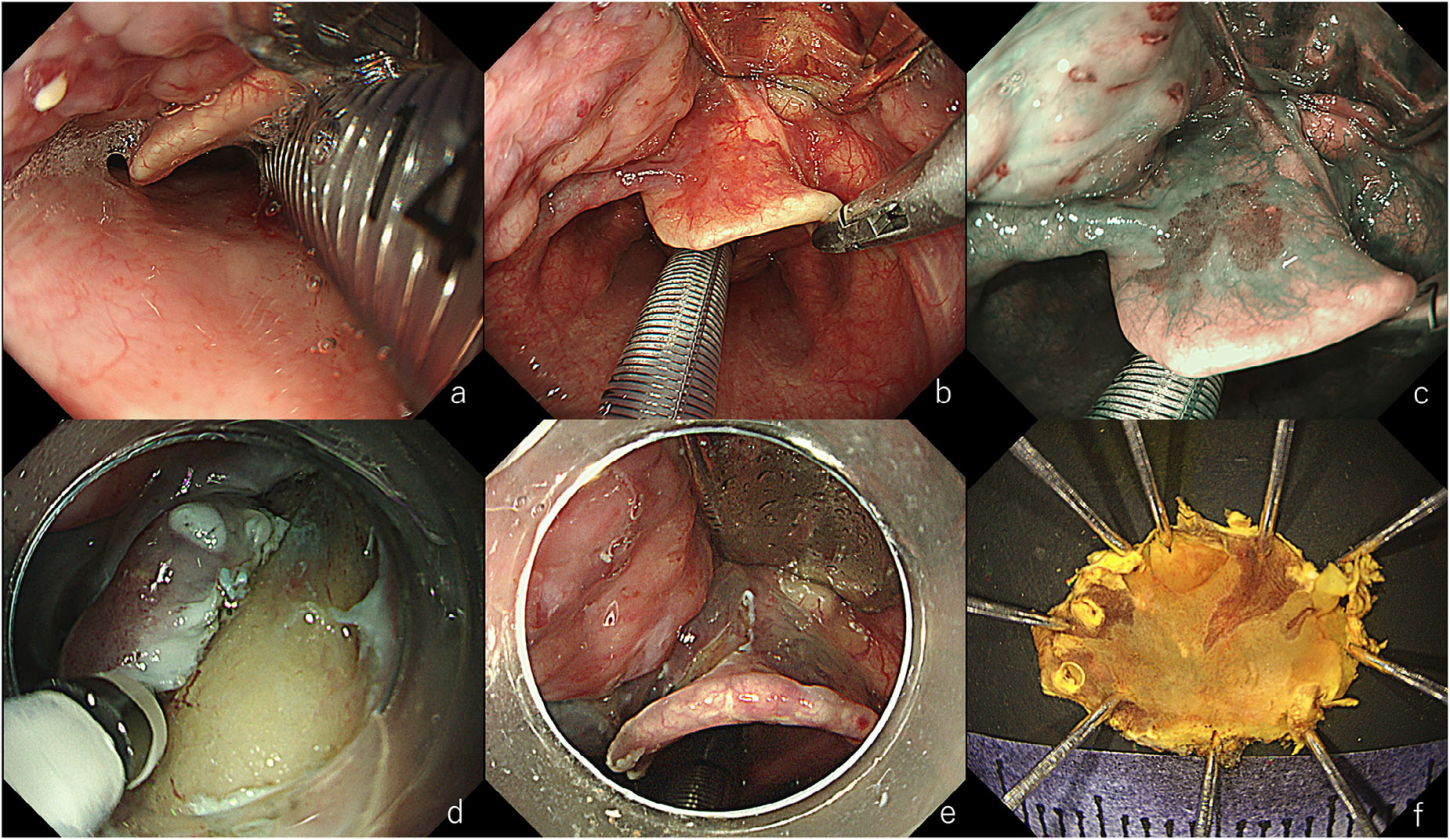
Endoscopic submucosal dissection (ESD) for a lesion in the vallecula. (a) Under general anesthesia, tongue depression caused poor visualization. (b) Nasal intubation positioned the tube along the posterior wall, facilitating a better view. (c) A clearly defined brownish area is recognized using narrow‐band imaging (NBI). Retraction of the epiglottis using laryngeal forceps improves visualization. (d) During dissection. A thin endoscope is useful for dissection in a narrow space. (e) Post‐resection ulcer base. Cartilage was exposed but without any injury. (f) Resected specimen. En bloc resection was achieved. Pathology revealed squamous cell carcinoma, 8×6 mm, tumor thickness 400 µm, ly0, v0, HM0, VM0.

**FIGURE 8:**
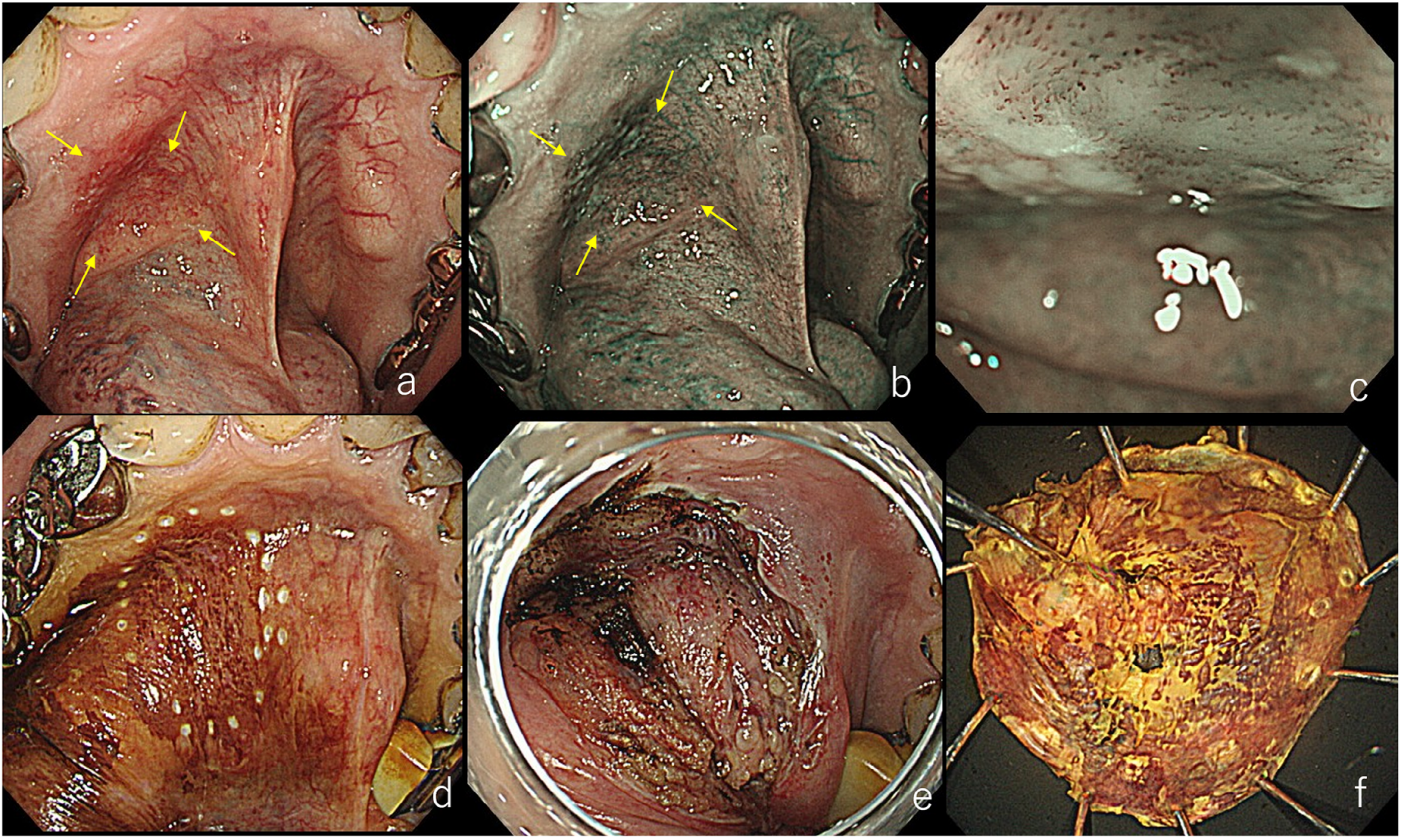
Endoscopy‐assisted tumor resection for a lesion in the floor of the mouth. (a) Slight erythema observed on the left floor of the mouth (yellow arrow). (b) The lesion appears as a brownish area, facilitating its detection (yellow arrow). (c) Dot‐like intrapapillary capillary loops (IPCLs) are proliferating, consistent with Type B1 vessels. (d) During marking. Spraying iodine solution revealed an unstained area. (e) After resection. (f) Resected specimen. Pathology revealed squamous cell carcinoma, 15×15 mm, SEP tumor thickness 220 µm, ly0, v0, HMX, VM0.

**FIGURE 9:**
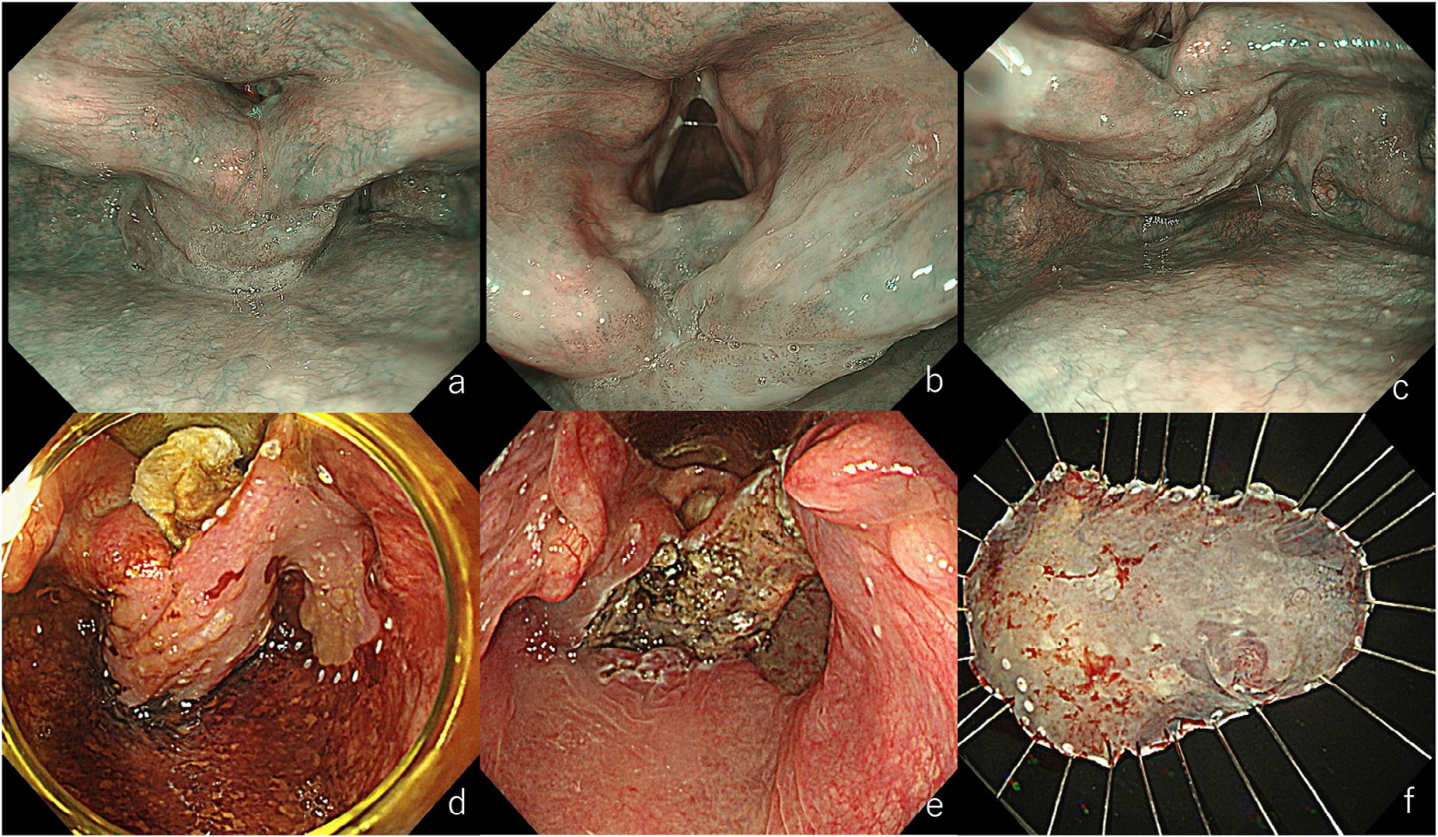
Tracheostomy‐assisted endoscopic submucosal dissection (ESD) for a lesion extending from the cricoid area to the right piriform sinus. (a) Slight brownish area observed in the cricoid area. (b) Brownish area extending to the anterior commissure of the vocal cords. (c) During the Valsalva maneuver. Laryngeal elevation facilitates assessment of the extent of the brownish area. (d) During ESD marking. Tracheostomy and extubation were performed, and ventilation was managed through the tracheostomy. Marking up to the anterior vocal cords was achieved. (e) After ESD. Although laryngeal edema was present, respiration was unaffected due to the tracheostomy. (f) Resected specimen. Pathology revealed squamous cell carcinoma, 58 × 39 mm, SEP tumor thickness 527 µm, ly0, v0, HMX, VM0.

When lesions are located in difficult regions, alternate approaches may be more appropriate [28]. For lesions in the vallecula (Figure 7), nasotracheal intubation is preferred, and the tip of the laryngoscope is positioned at the base of the tongue to elevate the larynx. Lesions involving the soft palate, palatine arches, uvula, floor of the mouth, or tongue are best approached using a mouth gag to secure an adequate visual field (Figure 8). Marking and mucosal incision are performed endoscopically, while resection is safely and efficiently completed by an otolaryngologist under direct vision using electrocautery. The most technically challenging cases involve lesions extending from the aryepiglottic fold to the anterior vocal cords (Figure 9). In such cases, the endoscope cannot access the lesion due to the presence of the endotracheal tube. To overcome this, a tracheostomy is performed to remove the tube and create sufficient working space in the anterior glottic area. In narrow areas, the use of ultrathin endoscopes and tapered‐tip hoods may also facilitate the procedure.

### 4.4 Complications of ESD

Pharyngeal ESD is generally considered a relatively safe procedure with a low incidence of adverse events. However, several potential adverse events should be explained to the patient in advance.

- Perforation: The pharynx is surrounded by thick muscles and supportive connective tissues, which makes the risk of perforation relatively low. However, damage to the muscular layer may lead to subcutaneous emphysema. If secondary infection occurs, there is a potential risk of abscess formation.
- Bleeding: Intraoperative bleeding during pharyngeal ESD is rare. However, postoperative bleeding—particularly after extubation—can pose a risk of airway obstruction and must be clearly explained to the patient beforehand. In cases where endoscopic hemostasis is unsuccessful, emergency interventions such as tracheostomy may be required. Extra caution is warranted in patients receiving antithrombotic therapy.
- Pneumonia: In pharyngeal ESD, the risk of pneumonia is relatively higher than in other types of ESD due to exposure to irrigation fluids and blood during the procedure. Additionally, the risk of aspiration increases after resuming oral intake, making postoperative pneumonia a relatively common complication. Most cases respond well to antibiotic therapy. However, in patients with a high risk of aspiration, prophylactic tracheostomy may be considered.
- Pain: Postoperative pain is observed in nearly all patients following pharyngeal ESD. In some cases, opioid analgesics such as morphine are required for adequate pain control. The need for opioid use tends to be higher in cases with large tumor size or lesions located in the pyriform sinus.
- Functional Impairment: Unlike other anatomical regions, pharyngeal ESD carries the potential risk of affecting swallowing and phonation. These impairments may occur either in the immediate postoperative period due to laryngeal edema or later as a result of scar contracture. Although significant deterioration in the patient's quality of life (QOL) is uncommon, it is not negligible and should be thoroughly discussed with the patient prior to treatment.
- Laryngeal Edema: Laryngeal edema may occur as a result of local injection during ESD. In some cases, this edema prevents immediate extubation after the procedure, requiring delayed extubation on the following day after the swelling subsides. Reducing the volume of local injection can help minimize the risk of postoperative laryngeal edema.
- Dental Injury: During general anesthesia and laryngeal elevation, there is a risk of anterior tooth injury caused by the laryngoscope. If the patient has loose teeth, a preoperative dental consultation should be arranged to assess and address potential risks.

### 4.5 Post‐ESD Surveillance

With various technical refinements, the treatment outcomes of superficial pharyngeal cancer have become favorable [28]. In our institution, the en bloc resection rate and R0 resection rate as short‐term outcomes are 99.4% and 82.6%, respectively. When appropriate post‐ESD surveillance is conducted, the 5‐year overall survival rate is 84.1%, and the disease‐specific survival rate is 100%, indicating excellent long‐term outcomes. Therefore, appropriate surveillance should be performed after ESD for pharyngeal cancer. This should be considered from two perspectives: the risk of lymph node metastasis and the risk of metachronous carcinogenesis.

### 4.6 Pathological Evaluation and Additional Treatment

Unlike other early gastrointestinal cancers, the depth of invasion in pharyngeal cancer is assessed based on tumor thickness, measured from the surface to the deepest point of the lesion. A tumor thickness of ≥1000 µm and the presence of lymphovascular invasion are considered risk factors for lymph node metastasis [29, 30]. There is currently no established evidence regarding the necessity of adjuvant chemotherapy or chemoradiotherapy for patients with a high risk of metastasis. In pharyngeal cancer, metastases tend to remain localized to the cervical lymph nodes. Therefore, in cases where the vertical margin is negative, we generally opt for careful observation without immediate additional treatment. If the vertical margin is positive and there is a high risk of residual tumor, adjuvant radiotherapy may be considered.

### 4.7 Surveillance for Lymph Node Metastasis

Following pharyngeal ESD, surveillance for lymph node metastasis is conducted using contrast‐enhanced CT and cervical ultrasonography two to three times per year. When lymph nodes larger than 10 mm or nodes showing progressive enlargement are identified, PET‐CT is performed for further evaluation. Particular attention should be paid to lymph nodes on the same side as the resected lesion. If metastatic lymph nodes are suspected, pathological confirmation should be obtained. When the metastatic nodes are resectable, surgical lymphadenectomy is performed. If extranodal extension is confirmed on histopathological examination of the dissected nodes, postoperative radiotherapy is indicated. In cases where metastatic nodes cannot be surgically removed due to invasion into surrounding vessels or other structures, chemoradiotherapy is considered. With appropriate treatment and surveillance, patients with pharyngeal cancer initially treated by ESD generally have an excellent prognosis.

### 4.8 Surveillance for Metachronous Cancers

Squamous cell carcinoma is known for its tendency to develop as multiple lesions over time. Even after successful endoscopic resection, early detection and treatment of metachronous cancers are critical for achieving favorable long‐term outcomes [31, 32, 33]. We perform surveillance endoscopy twice a year. The incidence of metachronous cancer reaches 20%–25% at 5 years and exceeds 40% at 10 years, indicating that the risk does not diminish over time [31]. Therefore, long‐term surveillance is essential. At the time of initial ESD, iodine staining should be applied to the entire pharynx to stratify the risk of metachronous lesions. A greater number of unstained areas on initial iodine staining is associated with a higher risk of future cancers.

### 4.9 New Approaches to Surveillance

**FIGURE 10:**
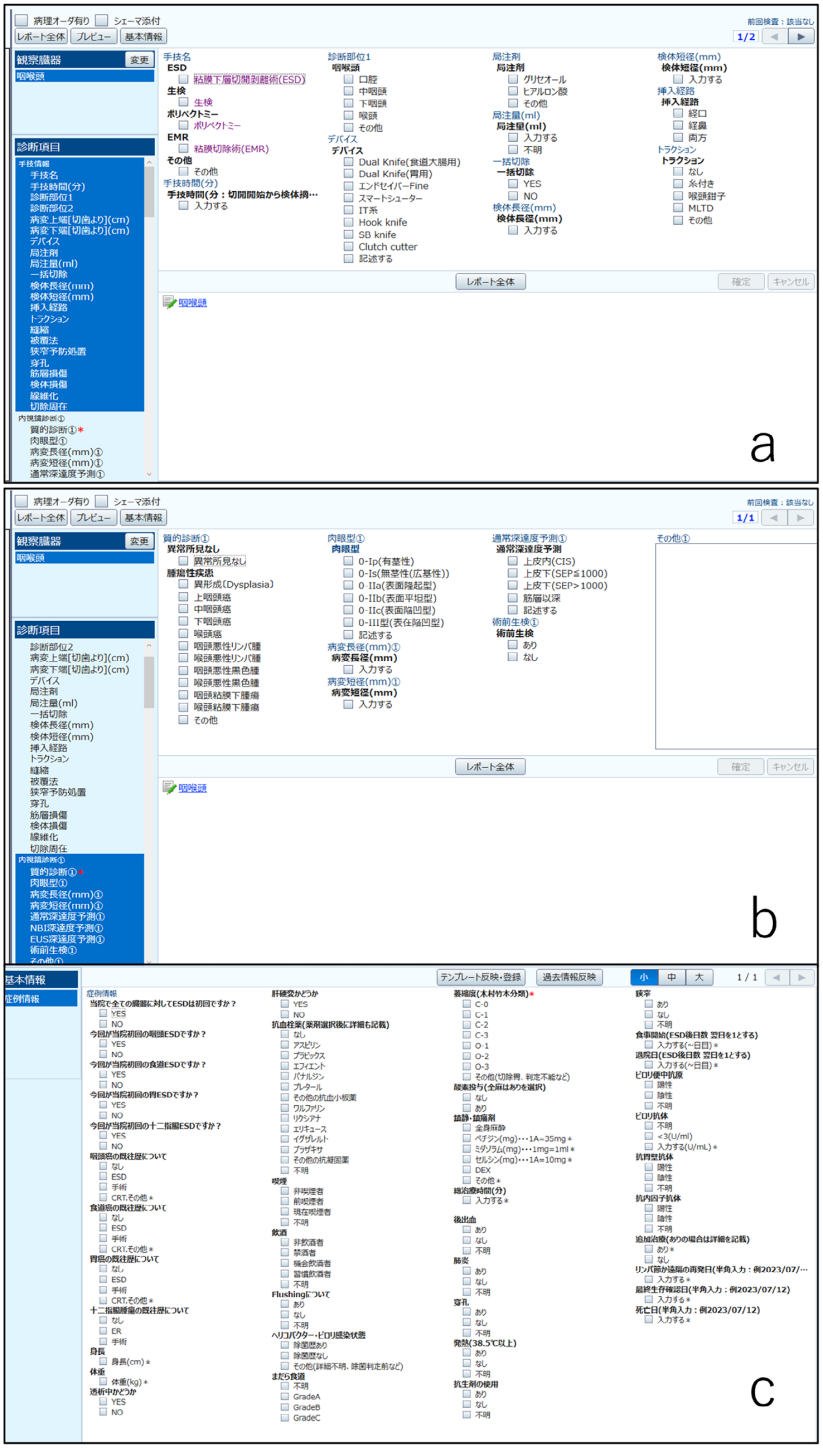
Efforts to establish a simple and accurate endoscopic submucosal dissection (ESD) database. (a) The data entry method was changed from entering endoscopic diagnosis first to entering procedural information first. (b) Up to three endoscopic diagnoses can now be entered following procedural information. Pathology results are linked to each endoscopic diagnosis. (c) A patient information sheet was created to enable per‐patient analysis. Using this input method, it became possible to construct an ESD database while conducting daily clinical practice.

Accurate construction of ESD databases for cancer types such as pharyngeal squamous cell carcinoma—which have high rates of metachronous and synchronous tumors—is inherently challenging (Figure 10). Due to the favorable prognosis of superficial pharyngeal cancer treated with ESD, long‐term follow‐up is required, and compiling comprehensive follow‐up data demands significant effort. ESD databases are often independently maintained by individual endoscopists, rather than being integrated into formal medical records, and there remains room for improvement in terms of accuracy and usability.

We have refined our documentation system for ESD to support per‐patient, per‐procedure, and per‐lesion analysis. Furthermore, we developed a system in which each surveillance endoscopy is linked to the corresponding ESD record, allowing for automatic confirmation of patient survival status. Looking forward, large‐scale analysis using the Japan Endoscopy Database is expected to play an important role. To facilitate such analyses, treatment endoscopy records—such as those for ESD—should be entered in a format optimized for structured data analysis.

## 5 Conclusion

This article has reviewed the current status of endoscopic management of superficial pharyngeal cancer. Early detection is of utmost importance and requires a clear understanding of the associated risk factors. Once detected, accurate diagnosis enables treatment that preserves the patient's QOL. Even after successful resection, appropriate follow‐up focused on both lymph node metastasis and metachronous cancer is essential to ensure favorable outcomes. Endoscopists play a central role in all aspects of care—detection, diagnosis, treatment, and surveillance—and are considered key contributors to effective multidisciplinary management of superficial pharyngeal cancer.

## Conflicts of Interest

The authors declare no conflicts of interest.
